# Honey Bee Exposure to Pesticides: A Four-Year Nationwide Study

**DOI:** 10.3390/insects10010013

**Published:** 2019-01-08

**Authors:** Nancy Ostiguy, Frank A. Drummond, Kate Aronstein, Brian Eitzer, James D. Ellis, Marla Spivak, Walter S. Sheppard

**Affiliations:** 1 Department of Entomology and the Center for Pollinator Research, The Pennsylvania State University, University Park, PA 16802, USA; 2 School of Biology and Ecology, University of Maine, Orono, ME 04469, USA; fdrummond@MAINE.EDU; 3 Honey Bee Breeding, Genetics and Physiology Research Unit, USDA-ARS, Baton Rouge, LA 70820, USA; katearonstein@gmail.com; 4 Department of Analytical Chemistry, The Connecticut Agricultural Experiment Station, New Haven, CT 06511, USA; brian.eitzer@CT.GOV; 5 Entomology and Nematology Department, University of Florida, Gainesville, FL 32643, USA; jdellis@ufl.edu; 6 Department of Entomology, University of Minnesota, St. Paul, MN 55108, USA; spiva001@umn.edu; 7 Department of Entomology, Washington State University, Pullman, WA 99164, USA; shepp@wsu.edu

**Keywords:** honey bees, pesticides, exposure, insecticides, herbicides, fungicides, pollen, wax

## Abstract

Pollinators, including honey bees, are responsible for the successful reproduction of more than 87% of flowering plant species: they are thus vital to ecosystem health and agricultural services world-wide. To investigate honey bee exposure to pesticides, 168 pollen samples and 142 wax comb samples were collected from colonies within six stationary apiaries in six U.S. states. These samples were analyzed for evidence of pesticides. Samples were taken bi-weekly when each colony was active. Each apiary included thirty colonies, of which five randomly chosen colonies in each apiary were sampled for pollen. The pollen samples were separately pooled by apiary. There were a total of 714 detections in the collected pollen and 1008 detections in collected wax. A total of 91 different compounds were detected: of these, 79 different pesticides and metabolites were observed in the pollen and 56 were observed in the wax. In all years, insecticides were detected more frequently than were fungicides or herbicides: one third of the detected pesticides were found only in pollen. The mean (standard deviation (SD)) number of detections per pooled pollen sample varied by location from 1.1 (1.1) to 8.7 (2.1). Ten different modes of action were found across all four years and nine additional modes of action occurred in only one year. If synergy in toxicological response is a function of simultaneous occurrence of multiple distinct modes of action, then a high frequency of potential synergies was found in pollen and wax-comb samples. Because only pooled pollen samples were obtained from each apiary, and these from only five colonies per apiary per year, more data are needed to adequately evaluate the differences in pesticide exposure risk to honey bees among colonies in the same apiary and by year and location.

## 1. Introduction

Pollinators, including honey bees, are responsible for the successful reproduction of more than 87% of flowering plant species: therefore, they are vital to ecosystem health and agricultural services world-wide [1]. Agriculture depends strongly on the services of the honey bee, which is responsible for providing more than 90% of commercial pollination services [2]. Thus, elevated loss rates seen in managed honey bee colonies [3] threatens the pollination services they provide. A variety of different stressors—including pathogens, parasites, floral resource loss, environmental stresses, and socioeconomic factors—have been suggested as contributing to the elevated levels of bee population decline [4,5,6,7,8,9,10,11,12,13]. One stressor, exposure to pesticides, has been, and remains, an important focus for research.

Until the early 1980s, the primary, if not exclusive, exposure to pesticides experienced by honey bees, and the source of hive product contamination, was from pesticides applied to agricultural or horticultural landscapes. With the introduction, nearly three decades ago, of the parasitic *Varroa* mite (*Varroa destructor*) into North America, concerns about pesticide residues in wax, honey, bees, pollen, bee bread, and other honey bee-related matrices expanded to include residues resulting from direct application of pesticides within colonies [14,15,16,17,18,19,20,21,22,23,24]. The most frequently reported compounds in honey bee products are acaricides used to control *V. destructor*, including coumaphos, fluvalinate, and amitraz; however, many other compounds have been detected, including some at levels that may result in sublethal and lethal outcomes [20].

To date, studies of pesticide residue in honey bee colonies have been cross-sectional. Residues have been evaluated in a variety of matrices, including but not exclusive to honey, beebread/pollen, wax, propolis, and bees [15,16,17,18,19,20,22,23,24,25,26,27,28,29,30]. These cross-sectional studies have described contaminants from samples obtained from multiple colonies/hives at a single time during the foraging season, or after the application of an acaricide to colonies. Most have investigated a limited range of pesticide residues, those primarily resulting from the management of honey bee pests [15,16,17,23,24,29,30]. Some studies used analytical methods capable of detecting of a wide range of pesticide residues found in the foraging environment as well as those used in colony management [18,19,20,22,25,26,28,31].

Mullin et al. [22], in their cross-sectional study of North American apiaries, detected 121 different herbicides, fungicides and insecticides, along with metabolites, in multiple matrices, e.g., wax, pollen, bee bread, and bees. In 259 wax samples, 87 compounds were detected with a mean of eight detections per sample. One sample contained residues of 39 compounds. The four most frequently detected pesticides in wax were fluvalinate (98.1%), coumaphos (98.1%), coumaphos oxon (89.9%, a metabolite of coumaphos) and chlorpyrifos (63.2%). Of these, fluvalinate, coumaphos, and coumaphos oxon are all compounds used by beekeepers against *Varroa*. In 350 pollen samples, a combination of bee bread (320 samples), trapped pollen (28 samples) and anther pollen (2 samples), 98 compounds were detected with a mean of 7.1 detections per sample. One sample contained residues of 31 compounds. The most frequently detected compounds were fluvalinate (88.3%), coumaphos (75.1%), and chlorpyrifos (43.7%), again with fluvalinate and coumaphos likely being introduced as compounds targeting *Varroa*.

Cross-sectional studies like these provide valuable information on bee colonies at a single point in time, or immediately following a single exposure. They are not capable of assessing the impact on bee health from multiple exposures, the cumulative and possibly synergistic effects of multiple sequential or simultaneous exposures, or the effect of multiple residues within a single matrix or spread throughout multiple matrices (e.g., wax, pollen, bee bread, nectar, honey, propolis, and adult bees, within a hive) [8,32]. The order of exposure is also important: Johnson et al. [8] demonstrated that the toxicity of amitraz was not changed when honey bees were exposed to sublethal doses of tau-fluvalinate or coumaphos prior to amitraz exposure, but tau-fluvalinate and coumaphos toxicity increased when bees were treated with amitraz prior to tau-fluvalinate or coumaphos.

This study is the first to sample the same colonies, from the time of their establishment until their death, using a prospective study design. Its objective is to extend the study of honey bee health impacts by assessing the level of pesticide contaminants, over a period of four years, carried to honey bee colonies by foragers in pollen loads. Colonies established in six states were included in the study.

## 2. Materials and Methods

### 2.1. Apiary Establishment and Management

Three trials (2009, 2010, and 2011) of stationary hives in six states were established for this study. In 2009, six apiaries with 30–32 colonies each were established. Three-pound packages were obtained from sources in the southeastern and western U.S. and introduced into new 10-frame wooden hive boxes (all deep boxes, except in PA, which used medium boxes) using Pierco^®^ wax-coated plastic foundation (Riverside, CA, USA). All Pierco^®^ wax-coated plastic foundation was purchased at the same time and the wax used for coating the foundation was from the same lot number (one wax source). Queens accompanying the packages were replaced with Italian queens from Koehnen & Sons, Inc. (Glenn, CA, USA).

In 2010 an additional 15-colony apiary was established in Orono, ME with packages originating from Florida. As in 2009, Italian queens from Koehnen & Sons, Inc. replaced the package queens.

In 2011, six apiaries with 30 colonies each were again established using 3-pound packages and new equipment as described above. Carniolan queens replacing those accompanying the packages were obtained from Koehnen & Sons, Inc. Because insufficient supplies of the Pierco^®^ wax-coated plastic foundation from 2009 were available, additional Pierco^®^ wax-coated plastic foundation was purchased in 2011 and the wax used for coating the foundation was from the same lot number (one wax source).

The habitat surrounding the apiaries varied from 68% to 4% agriculture (vegetable, fruit and field crops) and from 35% to 0% residential suburban. The general climate varied from the hot, dry summers and cold winters of eastern Washington, to the hot, humid summers and mild to cool winters of southern Texas, to the short warm, humid summers and cold winters of Minnesota, and the long hot humid summers and short cool winters of Florida.

Colonies were placed at least 2 m apart, where possible, in each apiary and managed in a manner typical for each region of the country (supering when necessary, wrapping in cold climates, etc.) except that *no pest management practices were employed*: i.e., no miticides, no medications for pathogens, no colony splitting resulting in intentional broodless periods, no screened bottom boards, no sugaring for mite control, and no drone frames were used during the lifespan of the colonies during the study.

Table 1 summarizes the location and location characteristics, year of establishment, number and survivorship after one year, of the study colonies in each apiary.

### 2.2. Pollen and Wax Sample Collection

#### 2.2.1. Pollen Samples among Apiaries and over Time

In all 4 years of the study (2009–2012), pollen samples were collected at each of the six apiary sites from the same five, randomly selected, living colonies every two weeks during the foraging season. If a colony died, another colony was randomly selected to replace it. Samples were collected every two weeks using a pollen trap operating for a brief period. Due to inclement weather (resulting in poor or non-existent foraging), or fungal growth resulting in pollen degradation, some locations did not have pollen samples for every two-week sampling period. The length of the foraging season varied by location. Florida (FL) and Texas (TX) had the longest season, with collection occurring from January/February through November/December. Pennsylvania (PA) and Maine (ME) had the shortest foraging seasons, with collection taking place from May/June through August/September. After each collection, pollen was sent to The Connecticut Agricultural Experiment Station (CAES) for pesticide residue analysis. Samples from each apiary were pooled by CAES for each bi-weekly collection. A total of 168 pooled samples were analyzed.

To evaluate if compounds detected in a colony are predictive of compounds likely to be detected in another colony, from 15 June–21 July 2009, 20 additional pollen samples were taken from five hives located in Washington (WA). A total of 20 unpooled samples were analyzed.

Table 1 includes a summary of the sampling at each apiary by year. The 20 unpooled samples from WA are noted separately.

#### 2.2.2. Wax Comb and Foundation Samples among States and over Time

##### Foundation

In 2009, prior to the installation of foundation in hives, two samples (minimum of 25 g/sample) were taken from randomly selected frames Pierco^®^ wax coated plastic foundation. These samples were sent to the CAES for pesticide analysis. In 2011, before packages were installed in the hives, three composite wax samples were obtained as described above from the unused Pierco^®^ wax coated plastic foundation purchased in 2009, and from Pierco^®^ wax coated plastic foundation newly purchased in 2011.

##### Wax Comb

In contrast to the pollen sampling, wax comb sampling was less frequent and not at consistent intervals. A total of 142 wax comb samples were taken. Most of these were obtained soon after colonies died, but not all hives in all apiaries were sampled after colony loss. A total of 107 wax comb samples were collected from dead colonies between 2010 and 2012 (FL (1), ME (8), MN (27), PA (16), TX (25), WA (30)). In addition, 35 samples of comb from living colonies were obtained in 2009, 2010, and 2012 (MN (5), WA (27), TX (3)). All wax comb samples were of 25 g or greater and taken from brood-rearing areas of hive. After each wax comb collection, samples were sent to CAES for pesticide residue analysis.

### 2.3. Pesticide Residue Quantification

#### 2.3.1. Sample Preparation

CAES performed the pesticide analysis for all wax and pollen samples. Except for the 20 pollen samples taken to assess the variability in compounds detected among individual colonies (see Section 2.2.1), all pollen analyses took place on pooled samples from each location. These pooled samples, one for each apiary and sampling period, were obtained by thoroughly mixing the samples from the five pollen-trapped colonies in each of the respective apiary. Pollen and wax samples were analyzed using a modified Quechers extraction protocol [25]. In brief, pollen samples (5 g if sufficient pollen was available or entire sample if less) were combined with water to a final volume of 15 mL. To this sample was added 100 ng of isotopically labeled (d-4) imidacloprid (Cambridge Isotope Laboratories Andover, MA, USA) as an internal standard. The samples were combined with 15 mL of acetonitrile, 6 g magnesium sulfate and 1.5 g sodium acetate, and 150 uL of acetic acid. After shaking and centrifuging, 10 mL of the supernatant was combined with 1.5 g magnesium sulfate, 0.5 g PSA, 0.5 g C-18 silica and 2 mL toluene. The samples were further shaken and centrifuged. Finally, 6 mL of the supernatant was concentrated to 1 mL for instrumental analysis. Wax samples were analyzed in a similar manner except that after addition of the water they were placed in a waterbath at 80 °C for 45 min to allow the wax to melt before shaking and centrifuging with the acetonitrile.

#### 2.3.2. Analysis

Extracts were analyzed with liquid chromatography/mass spectrometry/mass spectrometry (LC/MS/MS). In 2009, the LC system was an Agilent 1100 LC; 6 μL of the extract was injected onto a Zorbax SB-C18, 2.1 × 150 mm, 5 μm column. The column was gradient eluted at 0.25 mL per minute from 12.5% methanol in water to 100% methanol. Both solvents had 0.1% formic acid added. In 2010 the LC system was an Agilent 1200 Rapid Resolution system with a Zorbax SB-C18 (Agilent, Santa Clara, CA, USA) Rapid Resolution HT 2.1 × 50 mm, 1.8 μm column using a 3 μL injection with the gradient going from 5% methanol in water to 100% methanol at 0.45 mL/min. In both cases the LC was coupled to a Thermo-LTQ (Thermo-Fisher, Waltham, MA, USA, a linear ion trap mass spectrometer. The system was operated in the positive ion electrospray mode, with a unique scan function for each compound allowing for MS/MS monitoring. Detection limits varied with the exact sample matrix and the amount of sample available but were in the single digit part per billion (ppb) level or lower for pesticides reported herein.

Though our procedures were similar to those reported by Mullin et al. [22], we do not report the on the same set of pesticides as does that study. For enhanced specificity in pesticide detection at very low concentrations, we chose to use only our available LC/MS-MS. While providing greater confidence in our data on pesticides detected during a multi-residue screen, this choice precluded detection of some classes of pesticides (such as pyrethroid insecticides, also we could not detect chlorothalonil only its hydroxyl metabolite), which can require additional sample cleanup steps and GC/MS in electron impact or negative chemical ionization modes.

#### 2.3.3. Estimation of Oral and Contact LD_50_s

For the 82 pesticides (parent compounds), eight metabolites and one synergist detected in pollen and wax, we searched the literature for honey bee oral and contact LD_50_ [33,34,35,36,37]. Where LD_50_ values differed among literature sources, we used the value provided by the US EPA Ecotoxicity Database; if more than one LD_50_ value was reported in the EPA Ecotoxicity Database, we used the lowest value. Unless information specific to a metabolite was available, we used the LD_50_ value for the parent compound, acknowledging that some metabolites have lower toxicity than the parent compound and some have higher toxicity than the parent compound [34,38]. For 35 compounds only a contact LD_50_ was found; for three compounds only an oral LD_50_ was found. No contact or oral LD_50_ was found for two compounds (diphenylamine and octhilinone). Oral and contact LD_50_ values reported in terms of µg/bee were converted to ppb relative to body weight (ng pesticide per g bee) by multiplying each value by a factor of 10,000; this is equivalent to 1000 ng per µg, divided by a mean bee weight of 0.1 g [39].

### 2.4. Statistical Analysis

#### 2.4.1. Pollen, Comb Wax, and Foundation Wax

Goodness of Fit and Tests of Independence were conducted to determine similarities and differences between pollen and wax pesticide residues or oral versus contact LD_50_. Wax sample (comb and foundation) residues were logarithm +1 (base 10), transformed to achieve homogeneity and normality of variances. Pearson and Spearman’s correlation coefficients were used to assess evidence of association among types and classes of pesticide residues in detected pollen. An analysis of variance (RCBD) was used to determine if significant differences existed among residue concentrations in a paired study involving wax comb (near the brood rearing area) taken from hives with living colonies and hives where the colonies had died.

#### 2.4.2. Can Residues Detected in Pollen Samples a Colony Predict the Residues in Pollen in an Unsampled Colony?

To evaluate if compounds detected in a colony are predictive of compounds likely to be detected in another colony, from 15 June–21 July 2009, 20 pollen samples were taken from five hives located in WA. These samples were individually analyzed by CAES: i.e., they were not pooled before analysis. Each sample was either positive or negative for the presence of any detectable pesticide. To quantify the degree of variability we introduce the Predictive Power of individual pollen samples:

Predictive Power = (# of correct predictions)/(# of incorrect predictions).

To evaluate the (# of correct predictions) and (# of incorrect predictions), first pool the pesticide detections found, in any sample, at each sampling time. A correct prediction at a sampling time corresponds to a detection of a pesticide from that pooled list in an individual sample. For example, if—in a given sampling time—there were ten pesticides detected in five samples, the number of correct predictions at that sampling time would be at least ten, but no greater than fifty. The number of correct predictions is the total of correct predictions across all sample times less the number of pesticides detected.

Likewise, an incorrect prediction at a sampling time corresponds to a pesticide in the pooled list that was not found in one of the individual sample assays from which the list was assembled. Using the same example numbers, the number of incorrect predictions at a sampling time could be as low as zero, or as high as forty. The number of incorrect predictions is the total of incorrect predictions across all sample times.

It is important to note that, by our definition, a prediction occurs only when a pesticide is detected: i.e., the absence of a pesticide detection in a sample does not count as a prediction that the same pesticide will not be found in other samples taken at the same time. This choice was made to eliminate false negatives: i.e., situations in which the pesticide was not detected because the pesticide was not in the environment on the day the pollen was sampled. Subtracting the number of pesticides detected at each sampling time is necessary to eliminate the bias introduced by counting only pesticide detections as predictions.

As defined, a Predictive Power of unity means there is 50% chance of that a pesticide detection in a single hive correctly signals the presence of that pesticide in any other hive. A predictive power greater than unity corresponds to a greater than 50% chance that other hives will show presence of the same pesticide, while a predictive power less than unity means that detection of a pesticide in one hive is anti-correlated with the presence of the pesticide in other apiary hives.

## 3. Results

Table 2, Table 3, Table 4 and Table 5, provide different perspectives on the number of detections in the different matrices (pollen, wax comb, and foundation wax), the number of compounds detected in each matrix, and the years in which each compound was detected. Salient features of the data are described in the subsections that follow.

### 3.1. Pollen and Wax Pesticide Residues—General Findings

Viewed as a whole, 716 compound (pesticides, metabolites and/or synergists) detections occurred in the 168 pooled pollen samples and 993 detections occurred in the 142 wax comb samples. These 1709 detections were of 91 different compounds. Of the 91 compounds detected, 42% (38) were insecticides, 32% (29) were fungicides, 25% (23) were herbicides, and one was a synergist.

Of these 91 different compounds detected, 79 pesticides and metabolites were detected in the pollen samples, while 58 pesticides, metabolites and/or synergists were detected in wax.

Also, of the 91 compounds detected, 32 were found only in pollen, 12 were found only in wax, and 47 were found in both wax and pollen. If a pesticide or metabolite was found only in one matrix (pollen or wax), the pesticide or metabolite was significantly more likely to be found only in pollen rather than in wax (χ^2^_(1)_ = 11.5; *p* < 0.001).

Table 2, Table 3, Table 4 and Table 5 show all detected compounds, the matrix (pollen, wax comb, or foundation wax) in which the detections were made, and how many detections were made of each compound in each matrix. Table 2 organizes this information by pesticide and includes each pesticide’s oral and contact LD_50_, matrix and years of detection, mean concentration and range and mode of action (MOA) [40,41,42]. Table 3 organizes the residues in pollen by year. Table 4 organizes the wax comb residues by year and Table 5 describes the foundation wax residues.

### 3.2. Predictive Power of Pollen Samples from a Single Colony to an Apiary

Twenty pesticides were detected in the 20 unpooled pollen samples obtained from the five WA colonies. The mean (SD) number of residues detected per unpooled pollen sample was 6 (1.7); no sample had fewer than three detections. Dimethoate was detected in all samples. Acephate, carbaryl, chlorpyrifos, dithiopyr, epiconazole, and fluvalinate were each detected in a single sample.

The predictive power, defined in Section 2.4.2, of a pollen sample from one colony to indicate the presence of a pesticide in other colonies in the same apiary was 2.75: i.e., the presence (absence) of a pesticide in a pooled sample was a good predictor of the presence (absence) of the compound throughout the apiary.

### 3.3. Fungicides in Pollen, Wax Comb and Foundation Wax

Of the 1709 compound detections in pollen and wax, the number of fungicide and their metabolites detections in pollen, wax comb, and wax foundation were 192, 262, 24, respectively. These fungicide detections were of 29 different fungicides. Of these 29 fungicides, 24 (83% of all fungicides found) were found in pollen and 20 (69%) were found in wax. Also of these 29 fungicides, 9 (31%) were found only in pollen samples, while five (17%) were found only in the wax samples.

Table 3 summarizes the assay results for pesticides in pollen. Focusing on the 24 fungicides detected in the pollen samples, nine (38%) were detected in only a single year, while four (17%) were detected in all years. The fungicides carbendazim, azoxystrobin, and propiconazole-1 were the most frequently detected compounds in pollen: 48 (28% of the 168 samples), 41 (24%), and 32 (19%), respectively. The greatest number of fungicides detected in any one year was in 2011, when 17 different fungicides were detected. Also in 2011, the highest concentration of any single fungicide, carbendazim (548 ppb), was observed.

Table 4 summarizes the assay results for pesticides in wax comb samples. Focusing on the 14 fungicides detected in the wax comb samples, five (36%) were detected in only a single year while two (14%) were detected in all four years. The fungicides azoxystrobin and pyraclostrobin were the most frequently detected compounds in wax: 56 (39% of the 142 wax comb samples) and 53 (37%), respectively. The greatest number of fungicides detect in any one year was in fall 2010, when 11 different fungicides were detected. The highest concentration of any single fungicide in wax was 137 ppb of carbendazim in the (established Spring 2009)/(sampled Fall 2011) samples of drawn comb.

Table 5 summarized the results for pesticides in the wax used to coat the plastic foundation. Focusing on the 11 fungicides detected in the foundation wax, seven (64%) were detected only once and one fungicide, boscalid, was detected all five samples. Iprodione was found in the highest concentration—165 ppb.

### 3.4. Herbicides in Pollen, Wax Comb and Foundation Wax

In the 168 pollen samples, 142 wax comb samples, and 5 wax foundation samples, there were a total of 220, 166, and 8 detections of 23 different herbicides, respectively. Of the 23 detected herbicides, 20 herbicides were detected in pollen samples and 14 were detected in wax (comb and foundation) samples. Also of the 23 detected herbicides, 11 were found in both pollen and wax, nine (39%) were found only in pollen, and three (13%) were found only in wax.

Focusing on the herbicides detected in the pollen samples, 12 herbicides (60% of 20 herbicides detected in pollen) were detected only in a single year while four (20%) were detected in all 4 years. Atrazine was detected most frequently: it was found in 84 (50%) of the 168 pooled pollen samples. In pollen, the highest concentration of a single herbicide was of diuron (275 ppb) in 2011 (Table 3).

In comb wax, six herbicides (50% of 12 herbicides detect in wax comb) were detected only in a single year, while two, atrazine and diuron, were detected in all years and these two herbicides were the most frequently detected in the wax samples: atrazine was found in 49 (34%) of the 142 samples, and diuron in 41 (29%). In wax, the highest concentration of a single herbicide was of halosulfuron-mythyl (386 ppb) in 2010 (Table 4).

In foundation wax, only four herbicides were detected (Table 5). The herbicides at highest concentrations were pendimethalin and prodiamine (17 ppb).

### 3.5. Insecticides in Pollen, Wax Comb, and Foundation Wax

In the 168 pollen samples, 142 wax comb samples, and 5 foundation wax samples there were a total of 304, 481, and 38 detections of 38 different insecticides, respectively. Of the 38 detected insecticides, 35 (92%) were detected in pollen samples and 24 (63%) in wax (comb and foundation) samples. Also, of the 38 detected insecticides, 21 (55%) were detected in both pollen and wax, 14 (37%) were found only in pollen, two (5%) only in wax comb and one (3%) only in wax foundation (Table 2, Table 4 and Table 5). The synergist piperonyl butoxide was detected only in wax samples: 56 times in wax comb and 3 times in foundation wax.

Pollen sample assays detected 35 different insecticides. Of these, 12 (34% of insecticides detected in pollen) were detected only in a single year while six (17%) were detected in all 4 years. Carbaryl was most frequently detected insecticide: it was found in 26% of the 168 pooled pollen samples. Imidacloprid or its metabolites were detected only 11 times (6% of the 168 samples) and it was not detected at all in 2010. In pollen, the highest concentration of a single insecticide was of spinetoram (645 ppb) in 2011 (Table 3).

In comb wax, 22 different insecticides were detected (Table 4). Of these, 10 insecticides (45% of insecticides detected in wax) were detected only in a single year while four (18%) were detected in all years. Even though no miticides were used in the colonies in this study, coumaphos was the most frequently detected insecticide/miticide: it was found in 122 (86%) of the 142 wax comb samples and 100% of the five foundation wax samples. Fluvalinate, another insecticide/miticide approved for use in honey bee colonies but never used in this study, was the second most detected insecticide: it was found in 111 (78%) of the 142 wax comb samples (Table 4) and 100% of the five foundation wax samples (Table 5). A metabolite of amitraz, another miticide used to control Varroa mites, also was found in 100% of the wax foundation samples.

### 3.6. Annual Pattern of Residues

In all years, more types of insecticides were observed in pollen and wax than were types of fungicides or herbicides: 38 (42%), 29 (32%), and 23 (25%), respectively (see Table 2). In pollen, the greatest number of detections (223) and the greatest number of compounds (56) occurred in 2011. Also, in pollen, the smallest number of detections (138) and compounds (33) were found in 2009 (Table 3). In wax comb, the greatest number of detections (458 detections in 65 samples (7 detections/sample)) occurred in fall 2012 and the greatest number of compounds (37) were found in fall 2010. However, the greatest number of detections per wax comb sample occurred in fall 2012: 64 detections in 7 samples (9.1 detections/sample) (Table 4).

#### 3.6.1. Annual Pattern of Residues within Pollen Samples

The largest number of residues detected in a single pooled pollen sample was 10, 9, 13, and 12 compounds in 2009, 2010, 2011, and 2012, respectively. No detectable residues were found in eight of the 168 pooled samples: one from Maine in 2009, two each from ME and FL in 2011, and three from PA in 2012. The maximum number of compounds found in a single pollen sample was 13 (WA, 2011). The overall mean (SD) number of detections per sample was 4.1 (2.7). Table 6 gives the mean (SD) number of detections per pollen sample broken down by year. The mean (SD) number of detections per pooled pollen sample varied by location from a low of 1.1 (1.1) in ME in 2011 to a high of 8.7 (2.1) in TX in 2012.

Table 7 presents the three most common pesticides found in pollen samples for each year of the study (summarized from Table 3). Over the four years of the study, the herbicide atrazine was the most commonly detected pesticide; it was also the most frequently detected pesticide in each individual study year. Carbaryl, an insecticide, was among the three most frequently detected pesticides detected in years 2010 and 2012. It was also the third most commonly detected pesticide over the four study years. The fungicide carbenzadim made the “top three” most frequently detected pesticides in only one study year (2011); however, it was the second most commonly detected pesticide over the four study years.

Most pollen residues tended to be episodic—occurring in some but not all locations and in some but not all months or years. Thus, predicting the presence of a residue based upon prior detection was not possible. For example, imidacloprid was detected in PA in July and August of 2009, MN in August and September of 2011, TX in July and MN in August of 2012 but in no other locations or months/years. Tebuconazole was not detected in any of the six locations in 2009 and 2010. In 2011, it was detected once in TX in August, twice in WA in June and August and in 2012 it was found only in TX in March. Propiconazole in 2009 was observed in WA in two non-sequential months—June and August—but then was found in 2010 through 2012 from May–September. In ME, FL and MN propiconazole was only episodic. It was detected in ME in three months—May 2010 and June and July 2011 while in FL and MN the only detection was in November 2010 and August 2011, respectively. In MN and FL dimethoate was detected only in June 2009 and it was never detected in PA or ME.

While most pollen residues were episodic, in some locations a few residues occurred more continuously. Dimethoate was found in WA from May/June through September in all four years. Azoxystrobin was detected in WA in June and July 2009 and from May/June through September 2010, 2011, and 2012, and in MN from July through September 2011 and 2012. Atrazine was found in all locations but somewhat continuous only in MN, PA, and WA. It was detected in MN from May/June through September 2010, 2011 and 2012, in PA from June through August 2012, and in TX from May through December 2009, February through July 2010 and in March through July 2012.

#### 3.6.2. Annual Pattern of Wax Comb and Wax Foundation Residues

Table 4 summarizes detections in wax comb taken from active colonies, while Table 5 summarizes detections in foundation wax. A total of 58 different compounds were detected in these samples. More compounds were detected in drawn wax comb (49) than in wax-coated foundation (27). Not all compound detections in wax comb and wax foundation were the same. Thirty-three compounds were found only in wax comb and eight were found only in the foundation wax. Fifteen compounds (five fungicides, two herbicides, and eight insecticides) were found in both comb and foundation wax.

In drawn wax comb, there was a mean (SD) of 7.1 (1.7) compounds per sample, and a total of 49 compounds detected. Of the 49 detected compounds, 14 were fungicides, 22 were insecticides, 12 were herbicides, and one was a synergist. The greatest number of fungicides (11 of 14) and insecticides (19 of 22) detected in any single year were detected in the fall 2010 wax comb samples, while the greatest number of herbicides (8 of 12) detected in any one year was in the fall 2012 wax comb samples. The maximum number of residues per wax comb sample varied by location from three in FL to ten in TX.

When colonies were established in 2011, the wax used to coat the plastic foundation was from two different batches (lot numbers) of wax (see Section 2.1). Therefore, foundation wax samples from PA 2009 (purchased and sampled in 2009), PA 2011 (purchased in 2009 but sampled in 2011) and ME 2011 (purchased and sampled in 2011) were compared. There was a weak but positive association in contaminants between the samples from the same manufacturer but different batches; the PA 2009 and PA 2011 (same batch) and ME (different batch, same manufacturer); *r* = +0.433, *p* = 0.0273 and *r* = +0.326, *p* = 0.105; PA 2009 vs. ME 2011 and PA 2011 vs. ME 2011, respectively. There was a high correlation between the PA 2009 and 2011 foundation wax samples (*r* = +0.948, *p* < 0.001); they were from the same manufacturer and batch.

The foundation used to initiate the colonies in 2009, 2010, and 2011, was found to have 26 pesticides or metabolites with a mean (SD) of 14.2 (4.2) detections per sample. Of the 26 compounds detected in the foundation wax, 11 were fungicides, four were herbicides, 10 were insecticides, and one was a synergist. The number of detections per sample ranged from nine to 19. Two metabolites (amitraz DMPF and coumaphos oxon), two insecticides (fluvalinate and coumaphos), and one fungicide (boscalid) were found in all of the wax foundation samples. One herbicide (pendimethalin), one fungicide (azoxystrobin), and three insecticides (chlorpyrifos, fenproximate, and propargite) were found in 80% of wax foundation samples.

There were 16 contaminants found in common to both the wax comb and foundation wax (five fungicides, two herbicides, and eight insecticides/miticides, and one synergist).

In contrast to the pollen sampling, wax sampling was less frequent and not at consistent intervals (see Section 2.2.2). Whether the data were log transformed or not, there was no evidence of a correlation between the frequency of detection of insecticides in wax comb and foundation wax for compounds that were found in both in either 2009 or 2011 (*p* > 0.10). When data were pooled over years there was a weak correlation when the data were transformed (log ppb) between concentrations of pesticides in foundation wax and wax comb (*r* = +0.448, *p* = 0.054).

The contaminants in the wax foundation used to initiate the hives in 2009 was not correlated with the mean monthly, maximum, or total seasonal contaminants found in pollen brought into hives in 2009 for any of the six apiaries in the 2009 trial. Only three of the apiaries in 2009 shared some of the top five most abundant contaminants in pollen with those most abundant compounds in the foundation wax (fluvalinate in two apiaries and coumaphos in three apiaries).

### 3.7. Comparison of Wax Comb Residues from Living vs. Dead Colonies

Comparison of wax comb sampled from both living and dead colonies in the same apiary on the same date revealed that pesticide detection number did not differ (*p* > 0.10); however, the total pesticide concentration (sqrt ppb) showed a possible trend of lower total concentration in wax combs from living colonies than from wax comb from dead colonies (*F*_(1,6)_ = 5.3566, *p* = 0.06).

### 3.8. Lethal Dose Levels of Residues in Pollen and Wax Comb

Of the parent pesticides and metabolites for which both honey bee oral and contact LD_50_ values were available (51 compounds), 12 (24% of the 91 detected compounds) had the same LD_50_ values for the contact and oral mode of entry, 18 (35%) had lower oral than contact LD_50_ values (oral exposure was more toxic than contact exposure) and 21 (41%) had lower contact than oral LD_50_ values (contact exposure was more toxic than oral exposure). There is no evidence, based upon the oral versus contact LD_50_ values for the detected compounds, to suggest that, independent of quantity of pesticide used, oral exposure to a pesticide of a honey bee is more lethal than contact exposure (χ^2^_(2)_ = 2.0799; *p* = 0.35).

While some pesticides were frequently detected, most never were detected at a level that was lethal to honey bees. For example, the herbicide atrazine, was never detected at a level higher than 10^−6^ of its contact LD_50_. Likewise, the most frequently detected fungicide in pooled pollen was carbendazim, which was never detected higher than 0.1% of its contact LD_50_. Among the compounds detected in pollen, the greatest acute risks were posed by insecticides: while imidacloprid and its metabolites were infrequently detected in the pooled pollen samples (11 of 168 samples), in one sample imidacloprid was measured at 137% of its oral LD_50_. Another insecticide, dimethoate, was detected at near 35% of its oral LD_50_. The largest measured residue of two other insecticides, phosmet, and pyridaben, were 13% of their respective LD_50_s.

The herbicide, fungicide and insecticide residues in comb wax posed a lower acute risk than pollen. In wax comb, the highest concentration of a single fungicide was diphenylamine at 227 ppb, for which no oral or contact LD_50_ has been determined. No fungicide was detected in wax comb at a level greater than 0.2% of its LD_50_; no herbicide was found in wax comb at a level greater than 0.01% of its LD_50_. In wax comb the greatest risk was, again, for insecticide exposure: in 2010, 2011, and 2012, the maximum fluvalinate concentrations were 862 ppb, 512 ppb, and 537 ppb, respectively, which correspond to 43%, 25%, and 26% of fluvalinate’s 2000 ppb contact LD_50_.

In foundation wax the most acute risk was posed by the insecticide fluvalinate, which was measured at 7.7 times its LD_50_. Amitraz, because of its ability to interact with fluvalinate and coumaphos [8], likely also posed a significant risk (a metabolite of amitraz was detected at 582.3 ppb); however, its LD_50_, acting alone or in combination with fluvalinate and coumaphos, is unknown. Of the remaining pesticides, the insecticide phosmet, posed a low—though not negligible—risk with its largest measured residue level being 5% of its LD_50_. The variation among replicate samples of foundation, estimated by the coefficient of variation when including all 26 contaminants, was 105.6%. This level indicates the vast majority of the 21 contaminants have low mean concentrations.

### 3.9. Modes of Action Distributions in Pollen, Wax Comb, and Wax Foundation

Different pesticides may act on target organisms in similar or in different ways. Pesticides that act in the same way are said to be of the same class; pesticides that act in different ways are of different classes. The different classes of pesticides detected in pollen, wax comb, or foundation wax, are summarized in Table 8. Whether in pollen, wax comb, or foundation wax, a greater number of classes of insecticides (14) were observed than of fungicides (11) or herbicides (8). The fungicides that were detected ranged from benalaxyl, an inhibitor of nucleic acid synthesis to chlorothalonil, a multi-site activity fungicide. Herbicides detected varied from atrazine, an inhibitor of photosynthesis, to sethoxydim, an inhibitor of acetyl CoA carboxylase. Insecticides detected varied from spiriclofen, an inhibitor of acetyl CoA carboxylase (as is the herbicide sethoxydim) to imidacloprid, a nicotinic acetylcholine receptor agonist. Among the fungicides, the largest number of compounds (9) was found in class G (MOA: sterol biosynthesis in membranes). The herbicide group with the greatest number of compounds (10) was class C (MOA: inhibition of photosynthesis at photosystem II). Insecticide class 1B contained the greatest number (11) of compounds (MOA: acetylcholinesterase inhibition).

In pollen, the most frequently detected pesticide was the herbicide atrazine, a photosynthesis inhibitor. Of the insecticides detected in pollen, acetylcholinesterase inhibitors were detected in more samples than were any other class of insecticides. Among the fungicides, three compounds with different modes of action (B1, C2 and G1: see Table 5) occurred most frequently. If aggregated, the acetylcholinesterase inhibitors (239 detections) were detected more frequently in pollen than were the photosynthesis inhibitors such as atrazine (127 detections). If we suppose that each acetylcholinesterase inhibitor insecticide acts independently, then we might reasonably estimate the cumulative risk to this class of insecticides, in the “LD_50_” sense, as the sum of the ratios of each compound’s measured residue to its own LD_50_. If all acetylcholinesterase inhibitor insecticides exposure occurred simultaneously at the maximum detected level, then the total exposure for this class of insecticide would be 130% of the “net” acetylcholinesterase inhibitor LD_50_.

In wax comb and foundation wax, a greater number of classes of insecticides (10) were observed than classes of fungicides (9) and herbicides (7). Some modes of action were more commonly observed than others: the most frequently detected modes of action were the irreversible and reversible acetylcholinesterase inhibitors (358 of 559 insecticide detections, or 64%), followed by photosynthesis inhibitors such as atrazine (119 of 173 herbicide detections, or 69%), followed by respiration inhibitors such as azoxystrobin (131 of 275, or 48%, of fungicide detections) [40,41,42].

### 3.10. Potential Residue Interactions—Multiple Compounds

Linear correlations (co-occurrence) of pesticides in pollen were evaluated for pesticides of the same class, and between different pesticide classes. All correlations with individual *p* < 0.05 are reported in Table 9. Likely co-occurrences in pollen were observed between class B1 fungicides and C2 herbicides (*r* = 0.9660; *p* < 0.0001), between class C3 fungicides and class A herbicides (*r* = 0.9561; *p* < 0.0001), between class D1 fungicides and class C1 herbicides (*r* = 0.9955; *p* < 0.0001), between class C2 fungicides and class 28 insecticides (*r* = 0.9910; *p* < 0.0001), and between class A herbicides and class 28 insecticides (*r* = 0.9962; *p* < 0.0001).

## 4. Discussion

This is the only honey bee study to date to use a prospective study design sampling the same colony from the time of its establishment until its death. The colonies, once established, were not moved; thus the samples can be used to describe the temporal changes in pesticide residues in bee forage.

Differences in the number of compounds detected among Mullin et al. [22] and Stoner and Eitzer [25] and this study may be due to differences in the sampling protocols (same colonies sample throughout the foraging season and over multiple years versus single sample per colony), matrices sampled (pollen only versus pollen/bee bread and wax versus pollen and wax), time of year when sampling occurred, migratory versus stationary bee management, in-hive pest management methods, land use differences, length of time wax is in the hive, or the use of wax-coated plastic foundation or pre-drawn wax comb with pesticide residues. To fully characterize the exposure of honey bees to pesticides it is important to sample multiple colonies at multiple locations, with dissimilar management strategies, various colony longevities and different study methodologies.

A total of 91 pesticides, metabolites, and synergists from 335 pooled and individual pollen and comb and foundation wax samples, collected over four years, were detected in this study. Mullin et al. [22] detected 121 compounds in wax and pollen/bee bread while Stoner and Eitzer [25] detected 70 parent compounds and metabolites in pollen. A higher proportion (72% of 121) of pesticides in wax was reported by Mullin et al. [22] than in this study (48% of 91). It is not surprising that fewer compounds (44) were detected in 1 to 2-year-old wax from this study while a greater number of compounds (85) were reported by Mullin et al. [22] where some wax samples where older than 2 years. In this study atrazine, carbendazim, and carbaryl were the most frequently detected in pollen (48.6%, 27.7% and 25%, respectively). Mullin et al. [22] reported fluvalinate, coumaphos and chlorpyrifos (88.3%, 75.1%, and 43.7%, respectively) were the most frequently detected residues in pollen/bee bread and Stoner and Eitzer [27] reported coumaphos, carbaryl, and phosmet were most frequently detected residues in pollen (46.6%, 40.6%, and 32.9%, respectively).

The frequency of residue detection in pollen was similar between Stoner and Eitzer [25] and our study; <50% of the pollen samples were positive for at least one pesticide residue. This differed substantially from Mullin et al. [22], who reported 88.3% of the pollen samples positive for at least one pesticide residue. Differences in the types of compounds found in pollen and number of samples positive for residues may be due to an artifact of data reporting. Mullin et al. [22] reported pollen and beebread residues as a single category while our data and Stoner and Eitzer [25] are pollen samples collected from foragers prior to their entry into the hive. In-hive contamination of the beebread resulting from the use of amitraz, fluvalinate or coumaphos in the management of *Varroa* may explain the difference in the frequency of detection. The source of coumaphos found in the pollen analyzed in our study or by Stoner and Eitzer [25] is unknown. The pollen was never inside the colony, thus eliminating contamination by in-hive use of coumaphos, fluvalinate or amitraz. At this time there are no approved crop uses of coumaphos (the only approved use other than in honey bee management of *V. destructor* is as a cattle dip) unlike all the other compounds detected. As none of the pollen collected in this study or by Stoner and Eitzer [25] was ever in the colony, the source of coumaphos is of great interest. Possibly the source of coumaphos (and possibly fluvalinate and the amitraz metabolite) in the pollen in both our study and Stoner and Eitzer [25] could be due to residue transfer from the surface of the foragers’ body to the pollen. The source of surface contamination of the forager’s body could be bees from a package source where coumaphos was used, contamination picked up from wax residues in the colony or contamination of bee forage from unknown sources. Another potential source is the wax-coated foundation but a number of pesticides residues present in the wax-coated foundations samples were never detected in the drawn comb wax. A third possibility is that some coumaphos- or fluvalinate-contaminated pollen could have been obtained by bees stealing from honey bee colonies not a part of this project but located within flying distance to our apiaries. The remaining pesticides detected in pollen likely originated from pollen contamination from crop application.

Generally, insecticides were present more frequently and at higher concentrations than fungicides or herbicides. It would be interesting to explore the possible reasons for this result. One possibility would be to investigate potential correlations between prevalence of different types of pesticides to high versus low humidity conditions, differences in nighttime temperatures or the length of the growing season. Other possible explanations for the differences insecticide versus herbicide and fungicide detection that could be explored include similarities and differences in the chemical characteristics among insecticides, herbicides and fungicides, the types crops grown, regional differences in recommendations by Extension or pesticide company representatives and differences in the research emphasis by land grant universities.

The pattern of pollen and wax residues differed substantially by month and year. The number of detections was highest in 2011 and lowest in 2009. A compound may have been detected only once across all locations and years, at one or all locations but only once in a foraging season, multiple times (months) within one foraging season but at only one location or, at multiple locations or multiple years at multiple locations and multiple times (months) per foraging season. The differences by month, year and location are likely due to differences in target organism and the types of pesticides applied by growers, homeowners, and others to honey bee forage. Different pesticides may be used by growers because different crops have been planted, to address the occurrence of different pests or to reduce the development of pesticide resistance by pests. Differences among the apiaries may be a result of the surrounding land use or differences in climate. Two of the three apiaries surrounded by the highest concentration of agricultural land, TX and WA, tended to have the greatest number of residues and the most continuous exposure to the same compounds. Surprisingly, FL with its high humidity and long growing season had consistently fewer and more episodic detections than TX and WA.

Substantial variation in the quantity of residue detected in wax was found. Without information on the history of a wax sample, it is difficult to determine the reasons for the large variation in the quantity and types of pesticides detected. All the comb wax samples in this study were from stationary hives less than two years old, managed without the use of miticides or other pesticides and with a variety of environments surrounding the apiaries. Mullin et al. [22] obtained their samples from a variety of sources including colonies managed by small beekeepers, migratory beekeepers and commercial sources. Martel et al. [27] used commercially available wax. It is likely that the wax from these two studies included some old (>2 years) wax samples, samples from hives where beekeeper management likely included both registered and unregistered miticides, and wide-ranging surrounding environments. More studies with detailed information concerning the conditions experienced by the wax samples are needed to arrive at a clearer understanding of the presence and absence of compounds when studies are compared.

Interestingly, the type and the quantity of pesticides found in the same batch of wax used for coating foundation wax were not consistent. These data show that a better understanding of the dispersion of pesticide contaminants in wax is needed to explain the variation in types and quantities of compounds observed from the same batch of wax. Two likely reasons for the variation are the uneven distribution of residues in wax and the degradation of residues over time. Not knowing the causes of the variation nor the degree to which each cause influences the variation limit our ability to predict honey bee exposure.

Fewer studies of honey bee oral exposure to pesticides have been conducted than contact exposure. Of the 91 compounds detected in this study, two (2.2%) had neither an oral nor a contact LD_50_, two (2.2%) lacked a contact LD_50_ and 35 (38.5%) lacked an oral LD_50_ (Table 2). If the oral and contact LD_50_ for the remaining 52 compounds are compared, excluding the eight compounds with identical oral and contact LD_50_, the same number of compounds posed a greater oral exposure risk (lower oral LD_50_) as compounds posing a greater contact risk (lower contact LD_50_) (Table 2). Thus, the lack of oral LD_50_ studies may be less of a problem than expected.

Increased attention is needed on the MOA of various pesticides. Currently, the data on pesticide modes of action focus on the pests for which the pesticide has been developed and on resistance management for the target organism. Thus, important the modes of action for honey bees and other beneficial or non-target insects may be missed. An example is the herbicide atrazine with plants as the target organism; its published MOA is photosynthesis inhibition [41]. Atrazine has been studied in several non-target organisms including honey bees where the carotenoid-retinoid system is altered at exposure levels observed in agricultural systems. In rats, fish and quail the endocrine system is altered [43,44,45]. Thus, additional research is needed on pesticide modes of action for honey bee, other pollinators and non-target organisms. An additional area requiring research is, at minimum, to determine methods for summing exposures within the same MOA and how multiple modes of action may interact with one another.

Though the number of potential pesticide interactions and the varied types of target and non-target organisms is daunting, studies have been conducted on the combined impact from simultaneous and sequential exposure to pesticides [8,11,46,47,48,49,50,51,52,53,54,55,56,57]. Our study provides likely real-world examples of multi-residue exposure for future research. Because multiple residue exposure can result from the presence of more than one pesticide within a single pollen sample and from the all the residues in all pollen within a colony, it is important to gather data on pesticide residues in pollen and other hive products over multiple years and from many locations. One group of compounds found in our study to frequently co-occur was the reversible and irreversible acetylcholinesterase inhibitors and three of the miticides (amitraz, coumaphos, and fluvalinate) used for *Varroa* control. Other multiple exposures of interest, based upon pesticides detected in this study, include the possibility interaction of fluvalinate with resmethrin, the only other insecticide detected with the same MOA as fluvalinate, and the interaction of coumaphos with 10 other insecticides with the same MOA—acephate, azinphos-methyl, chlorpyrifos, diazinon, dichlorvos, dimethoate, malathion, omethoate, phorate, and phosmet.

Most efforts to understand pesticide interactions have focused on developing predictive models, not studies of specific compounds [49,50,51]. Due to the sheer number of potential combinations of pesticides, predictive models are a logical method to evaluate potential risk. Unfortunately, at least for our understanding of the risks to honey bees, the models have been built using common laboratory animal and plant model organisms, i.e., *Daphnia magna*, *Vibrio fischeri*, and *Stellaria media* [50]. Thus, the models are of little use to those attempting to predict real-world impacts of multiple pesticide exposure on honey bees.

Most of the pesticides detected in our study are for plant protection rather than honey bee colony management. Fungicides and herbicides may be applied to crops or horticultural plantings but would not be applied within a honey bee colony. Even among the insecticides detected, most are never used within the hive. Potential interactions between pesticides used and never used for colony management need to be explored along with interactions between pesticides only used for crop or horticultural plants.

Some studies focused on honey bees exposed to multiple pesticides have been conducted usually including at least one pesticide that may be used in honey be management. Colin and Belzunces [52], Pilling and Jepson [47], and Pilling et al. [48] explored the synergistic impact from paired pyrethroid and fungicide exposures. Sub-lethal exposures, either simultaneous or sequential, resulted in an increase in honey bee mortality; a reduction in the rate of pyrethroid biotransformation in the presence of the fungicide was proposed as the mechanism of action. More recently, Thompson and Wilkins [53] obtained similar results when they paired eight different fungicides and two pyrethroid insecticides but the evidence provided suggested a different mechanism of action. The presence of a fungicide decreased the repellency of the pyrethroid resulting in a higher exposure to the pyrethroid. Johnson et al. [8] reported that acaricides with different modes of action interact, usually resulting in decreased the LD_50_ compared to the single compound control in adult bees. Additionally, they showed that some fungicides interact with acaricides to lower the LD_50_ of the acaricide and that piperonyl butoxide lowers the LD_50_ of tau-fluvalinate. Data from our study show that fluvalinate and coumaphos could be found in the same sample of pollen, drawn comb, and wax-coated foundation and the piperonyl butoxide may co-exists with fluvalinate in wax.

Using honey bee larvae, Zhu et al. [54] tested fluvalinate, coumaphos, chlorothalonil, and chlorpyrifos alone and in combination. At moderate levels of exposure, the toxicity of fluvalinate-chlorothalonil and coumaphos-chlorothalonil increased the toxicity of the insecticides but at very low doses (10-fold reduction) the fluvaninate-chlorothalonil exposure became antagonistic. Complicating the picture further, when coumaphos was added to the fluvalinate-chlorothalonil mixture, the toxicity was reduced. The risk potential to adult and immature bees from pollen consumption, and to nurse bees, who recycle hive wax, and to new adult bees, who chew through wax to emerge from their brood cells, should be explored.

If pesticide exposure is evaluated using only the LD_50_ from each individual pesticide, based upon the results from this study, the risk posed to honey bees is low. In our pollen samples, one sample out of 168 exceeded the pesticide residue’s oral LD_50_. While much attention has been focused on the newer types of pesticides, ie. Neonicotinoids, and their impact on bee health, from this study it is clear than some of the older pesticides, specifically the carbamates and organophosphates, may continue to pose a risk. When the ratio of exposure (ppb) to its LD_59_ for the acetylcholinesterase inhibitors was summed, the ratio exceeded 1. While few samples had residues approaching the pesticide’s LD_50_, the pesticides residues detected are likely to pose a high sublethal risk to honey bee survival [7,46,47,48,52,53,54,55]. Currently it is difficult to evaluate the risks from sublethal exposures as there are no agreed upon outcome measures similar to the LD_50_ for the various sublethal outcomes. Additional work is needed, first, to define the most important sublethal outcomes and, second, to quantify the impact of sublethal exposures from various pesticides so a risk evaluation can be conducted.

## 5. Conclusions

Exposure, as estimated by pollen and wax comb contamination, is very complex and difficult to parse. Our study, and several others [22,25], has shown that many compounds at varying orders of magnitude of concentration are involved. In general, we found that insecticides/miticides were present more frequently and at higher concentrations than fungicides or herbicides. This was true in all four years. Our study also found that residues vary by month and by year making it difficult to generalize, without additional research, about which pesticides are most likely to be encountered by honey bees and which residues pose the greatest risk. Pollen was more contaminated than wax comb and foundation wax; one-third of the pesticides detected were only observed in pollen. We also found, on average, the potential oral toxicity of these residues was no greater than the potential contact toxicity. The modes of actions of the all pesticide residues in pollen and wax comb were diverse with 10 separate modes of action being found in all four years and nine additional modes of action occurring in only one year. If synergy in toxicological response is a function of simultaneous occurrence of multiple distinct modes of action, then we found high frequency of potential synergies in our pollen and wax comb samples. This prospective, longitudinal study expands our knowledge by providing pollen, wax comb, and wax foundation pesticide residue data throughout the lifespan of honey bee colonies in six locations across the U.S. over four years. (Approximately one-third of the 377 colonies in the study were sampled directly while the residues in other colonies were estimated indirectly using the predictive power—Section 2.4.2 and Section 3.2.) Previous residue analysis studies were cross-sectional, providing a single snapshot of honey bee exposure to pesticides. More data are needed to adequately evaluate the differences in pesticide exposure risk to honey bees, and other pollinators, by week, month, year and location; the pooled pollen sampling method used in this study did not provide the level of detail necessary to adequately describe the risk to honey bees and other pollinators.

## Figures and Tables

**Table 1:** Number of colonies alive (dead) by location and year including the number of wax and pollen samples. A pooled pollen sample is a composite bi-weekly sample of pollen collected from five colonies per apiary. The number of analyzed pollen samples reported in column 6 corresponds to pooled samples from five colonies in each apiary, except that analysis of unpooled samples, taken in 2009 from the WA apiary, is also reported in parentheses. The wax samples are comb wax; each sample is from a separate colony. See Section 2.1 (Apiary Establishment and Management) and Section 2.2 (Pollen and Wax Sample Collection) for further details.

		Initial # of Colonies	End of Year # of Colonies Alive (Dead)	# of Wax Samples	# of Analyzed Pollen Samples	Location	Surrounding Habitat
Florida (FL)	2009	30	22 (8)		8	Citra	25% agriculture * 19% forest, 38% pasture/grazing, 8% wetlands
	2010		6 (16)		12		
	2011	30	24 (6)	1	10		
	2012		0 (30)				
Maine (ME)	2009	30	26 (4)		6	Stockton Springs	30% agriculture, 35% forest, 20% shrub/old field, 8% wetland
	2010		10 (16)	2	4		
	2010	15	14 (1)		4	Orono	18% agriculture, 25% forest, 35% residential/suburban
	2011	30	28 (2)	6	7	Glenburn	30% agriculture, 35% forest, 20% shrub/old field, 8% wetland
	2012		25 (3)		8		
Minnesota (MN)	2009	32	31 (1)		5	Minnestrista	4% agriculture, 27% forest, 6% residential/suburban, 42% pasture/grazing, 20% other
	2010		20 (11)	4	5		
	2011	30	27 (3)	6	9		
	2012		6 (21)	22	10		
Pennsylvania (PA)	2009	30	27 (3)		3	Rock Springs	50% agriculture, 46% forest 4% shrub/old field
	2010		0 (27)	15			
	2011	30	27 (3)	1	4		
	2012		3 (24)		7		
Texas (TX)	2009	30	29 (9)	6	8	Weslaco	68% agriculture, 14% shrub/old field, 5% pasture/grazing, 13% other
	2010		3 (26)	2	11		
	2011	30	29 (1)	4	11		
	2012		7 (22)	16	10		
Washington (WA)	2009	30	29 (1)	20	4 (20 **)	Pullman	65% agriculture, 25% residential/suburban, 10% pasture/grazing
	2010		15 (14)	4	5		
	2011	30	27 (3)	6	10		
	2012		27 (0)	27	7		

* Agriculture land was vegetable, fruit and field crops. ** Twenty pollen samples were taken from five hives. These pollen samples were not pooled.

**Table 2:** Honey bee contact and oral LD_50_ for 91 compounds detected in pollen (P) wax comb (WC) and wax from coated foundation (WF). Mean, range, and LD_50_ values reported as ppb. The LD_50_ for the parent compound was used for a detected metabolite. Abbreviations: F = fungicide; H = herbicide, I = insecticide; U = unknown mode of action (MOA); nhbd = no honey bee data available. See Section 3.1 for details.

Pesticide	Detected in Pollen, Wax Comb or Wax Foundation	Pesticide Class MOA	Number of Detections: Pollen, Wax Comb, or Wax Foundation	Years Found: Detection Years	Detection		Oral LD_50_	Contact LD_50_	Source
					Mean	Range			
**FUNGICIDES**									
4-Hydroxy-chlorothalonil (chlorothalonil)	P		5	2009–2011	19.5	5.5–42		1,812,900	[35] *
	WC	Multi-site	7	2010	7.3	1.4–17			
Azoxystrobin	P		41	2009–2012	12.2	0.5–214	250,000	2,000,000	[35,37]
	WC	F-C3	56	2010–2012	2.8	0.5–33			
	WF		4	2009 & 2011					
Benalaxyl	P	F-A1	2	2010, 2011	1.4	1–1.9	1,000,000		[35]
Boscalid	P		10	2010, 2011	2.75	1.2–5.2	1,660,000	2,000,000	[35,37]
	WC	F-C2	14	2010–2012	19	0.6–43			
	WF		5	2009 & 2011	5.4	2.2–10			
Carbendazim	P		48	2009–2012	22.7	0.8–548		500,000	[35]
	WC	F-B1	40	2010–2012	61.5	0.7–137			
	WF		1	2009		1			
Cymoxanil	P	U	1	2010		7.5	852,900	250,000	[35,37]
Cyprodinil	P		1	2011		21		7,840,000	[35]
	WF	F-D1	3	2009 & 2011	49.4	8.7–90			
Difenoconazole	P	F-G1	2	2010, 2011	4.2	3.9–4.5	1,770,000	1,010,000	[35]
Dimethomorph	P		3	2009, 2011, 2012	0.55	0.5–0.6	324,000	100,000	[35,37]
	WF	F-H5	1	2011		3.9			
Diphenylamine	WC	U	3	2011	100.7	3.5–250	nhbd	nhbd	
Epoxiconazole	WC	F-G1	1	2010		0.5	830,000	1,000,000	[35]
Fenbuconazole	WF	F-G1	1	2009		2.5		2,920,000	[35]
Fluazinam	P	F-C5	1	2009		4.7	1,000,000	40,000	[35,37]
Fluoxastrobin	WC	F-C3	1	2012		13	8,433,000	2,000,000	[35]
Iprodione	WF	F-E	3	2009, 2011	133.3	110–165	250,000	2,000,000	[35]
Imazalil (Enilconazole)	P	F-G1	1	2009		1.6	351,000	390,000	[35]
Mandiproamid	P		1	2009		1.2	2,000,000	2,000,000	[35]
	WC	F-H5	1	2010		2.9			
Metalaxyl	P		4	2010, 2012	1.7	0.8–3.5	250,000	1,000,000	[35,37]
	WF	F-A1	1	2011		1.5			
Metconazole	P	F-G1	1	2011		1.3	850,000	1,000,000	[35]
Myclobutanil	P		2	2011, 2012	3.6	1.1–6.1		3,620,000	[25]
	WC	F-G1	2	2010, 2012	2.9	1.7–4.1			
Octhilinone	P		1	2011		24	nhbd	nhbd	
	WC	F-A3	7	2010, 2011	8	1.2–14			
	WF		1	2011		3.1			
Propiconazole-1	P		32	2009–2012	13.1	1.1–64	1,000,000	250,000	[35,37]
	WC	F-G1	36	2010–2012	6.3	0.8–66			
	WF		1	2009		2.6			
Pyraclostrobin	P		12	2010–2012	5.8	0.8–12	731,000	1,000,000	[35,37]
	WC	F-C3	53	2011, 2012	4.8	1.2–24.5			
Pyrimethanil	P	F-D1	1	2010		29	1,000,000	1,000,000	[35]
Spiroxamine	P	F-G2	2	2011	1.5	1.0–2.0	1,000,000	42,200	[35,37]
Tebuconazole	P		8	2011, 2012	65.6	2.0–104	850,500	2,000,000	[37]
	WC	F-G1	32	(2010–2012)	7.6	1.3–24			
Thiabendazole	P		2	2009, 2012	1.2	0.8–1.5	340,000	40,000	[37]
	WC	F-B1	9	2010–2012	46.1	0.4–91			
Thiophanate-methyl	P	F-B1	4	2010, 2011	13	4.6–40		1,000,000	[35]
Trifloxystrobin	P		7	2009–2012	6.8	0.2–23	2,000,000	2,000,000	[35,37]
	WF	F-C3	2	2009	1.8	1.3–2.4			
**HERBICIDES**									
Acetochlor	P	H-K3	4	2011, 2012	14.6	1.8–22		2,000,000	[35]
Alachlor	P	H-K3	1	2009		1.3		362,000	[35]
Atrazine	P		84	2009–2012	5.4	0.1–58		970,000	[33]
	WC	H-C1	49	2010–2012	0.9	0.01–6.5			
Bentazon	P		1	2010		3.1	2,000,000	2,000,000	[37]
	WC	H-C3	19	–2012	5.9	1.3–17			
Dithiopyr	P		3	2009, 2010	1.85	0.6–3		810,000	[35]
	WC	H-K1	4	2010–2012	2.4	1.7–5.1			
Diuron	P		27	2009–2012	19.5	1.1–275		1,450,300	[35]
	WC	H-C2	41	2010–2012	10.7	0.4–30			
Fenuron	P		2	2010	1.4	0.7–2.1		250,000	[35]
	WC	H-C2	2	2011	29.5	24–35			
Halosulfuron-methyl	WC	H-B	3	2010	307	213–386		1,000,000	[35]
Linuron	P		3	2010, 2011	5.2	2.2–14.4	1,600,000	1,208,600	[35]
	WC	H-C2	5	2010, 2011	2.4	1–5.5			
MCPA	WC	H-O	1	2012		4	100,000	250,000	[35]
Metolachlor	P		32	2009–2012	15.3	0.2–130	1,100,000	1,100,000	[27]
	WC	H-K3	12	2011, 2012	1.4	1–4.5			
Metribuzin	P	H-C1	3	2011	1.4	0.6–2.6	1660,000	604,000	[35,37]
Metsulfuron-methyl	P	H-B	1	2011		7	443,000	250,000	[35,37]
Napropamide	WC	H-K3	1	2011		2.8	1,000,000	1,000,000	[37]
Pendimethalin	P		41	2009–2012	6.6	1.2–55		498,000	[35]
	WC	H-K1	28	2010–2012	5.4	0.4–20			
	WF		4	2009, 2011	15.5	11–17			
Prodiamine	P		1	2009		2.3		1,000,000	[35]
	WF	H-K1	2	2009	15.2	13.4–17			
Prometon	P		3	2011	2.4	1–3.3		360,000	[35]
	WC	H-C1	1	2012		3.5			
	WF		1	2011		0.5			
Prometryn	P		1	2011		2.2		966,900	[35]
	WF	H-C1	1	2009		0.5			
Propyzamide	P	H-K1	3	2012	3.2	1.5–6.2		1,810,000	[35]
Sethoxydim	P	H-A	3	2010, 2011	13.4	1.75–23		100,000	[35]
Siduron	P	H-C2	3	2012	9.1	5.6–13		1,200,000	[35]
Simazine	P	H-C1	3	2009, 2011, 2012	1.5	1.1–2		967,000	[35]
Sulfentrazone	P	H-E	1	2011		2.5	250,000	250,000	[35]
**INSECTICIDES**									
Acephate	P		2	2012	2.4	1.6–3.2		12,000	[35]
	WC	I-1B	1	2010		5.8			
Acetamiprid	P	I-4A	3	2010, 2011	150.3	10–436	145,300	81,000	[35,37]
Aminocarb	P	I-1A	1	2011		1.7		600	[33]
DMPF (amitraz)	P		2	2010, 2011	2.5	2.5		1636	[35] *
	WC	I-19	2	2010, 2011	4.2	3.7–4.6			
	WF		5	2009, 2011	440.9	16.5–670			
Azinphos-methyl	P		3	2009–2011	1.7	0.4–3.5	1500	4200	[35]
	WC	I-1B	8	2010	0.3	0.2–0.3			
Bendiocarb	P		3	2010	1.7	1.2–1.9		4280	[35]
	WC	I-1A	1	2010		4.5			
Carbaryl	P		43	2009–2012	7.1	1–95	2310	11,000	[35]
	WC	I-1A	1	2010		0.9			
Carbofuran	P		6	2009–2011	7.9	1-24		1600	[35]
	WC	I-1A	10	2010	5.7	0.8–10.8			
OH-Carbofuran (carbofuran)	P	I-1A	6	2009, 2011	8.8	1.2–29		1600	[35] *
	WC		15	2010	28.1	5.4–112			
Chlorantraniliprole	P	I-28	2	2010, 2011	7.6	5.5–9.6	1,041,000	40,000	[37]
Chlorpyrifos	P		36	2009–2012	11	0.9–61	2500	100	[35]
	WC	I-1B	26	2010–2012	6.5	0.8–44			
	WF		4	2009, 2011	14.7	1-28			
Clothianidin	P	I-4A	6	2009, 2010, 2012	2	1.2–4.8	36.8	439	[25]
Coumaphos	P		31	2009–2012	10.2	0.2–196		240,000	[25]
	WC	I-1B	122	2010–2012	30	0.2–2060			
	WF		5	2009, 2011	1546.25	109–2500			
Coumaphos oxon (coumaphos)	P		1	2012		2.5		240,000	[25] *
	WC	I-1B	38	2010–2012	4.2	0.9–33			
	WF	=	5	2009, 2011	38.6	6.3–63			
Diazinon	P		4	2011, 2012	6.8	1–8.6	2000	2200	[35]
	WC	I-1B	11	2011	0.8	0.4–2			
	WF		1	2009		0.3			
Dichlorvos	P		2	2010, 2012	18	1.5–35		5000	[35]
	WC	I-1B	6	2010, 2011	3.6	2-13			
	WF		1	2011		10			
Diflubenzuron	P		1	2011		19			[35]
	WF	I-15	1	2011		3.8	351,000	390,000	
Dimethoate	P		27	2009–2012	23	0.3–194	560	1600	[35]
	WC	I-1B	47	2010–2012	23.9	0.6–945			
Fenpyroximate	P		2	2009	0.4	0.2–0.6	500,000	1,185,000	[35,37]
	WC	I-21A	10	2010–2012	23.2	1–207			
	WF		4	2009, 2011	98.4	4.1–366			
Fluvalinate	P		12	2009–2011	9.6	0.9–27		2000	[35]
	WC	I-3	111	2009–2012	157.5	1–862			
	WF		5	2009, 2011	8064.8	290–15,400			
Imidacloprid	P	I-4A	9	2009, 2011, 2012	7.4	1.5–51	37	439	[25]
Imidacloprid, Olefin	P	I-4A	1	2011		9.5	3600		[33]
Imidacloprid, urea	P	I-4A	1	2011		19	995,000		[33]
Malathion	P		33	2009–2012	65.8	1.4–148	3800	2000	[35,37]
	WC	I-1B	29	2010–2012	35.5	1.2–91			
Methiocarb	P	I-1A	7	2010–2012	3.7	1-20	3750	2300	[37]
Methomyl	P	I-1A	6	2011, 2012	10.4	2.5–26	2900	1600	[35]
Methoxyfenozide	P	I-18	4	2012	45.3	2.2–162	1,000,000	1,000,000	[35]
Omethoate (methoate)	P	I-1B	9	2011, 2012	4	1-12	560	1600	[35] *
Phorate	P	I-1B	1	2009		22	4400	3200	[35]
Phorate Sulfoxide (phorate)	P	I-1B	1	2011	3.3	1.7–9	4400	3200	[35] *
Phosmet	P		17	2009–2012	46.4	1.1–483	3700	10,600	[35]
	WC	I-1B	30	2010, 2012	15.8	1.1–220			
Propargite	WF	I-12C	4	2009, 2011	36.7	19–60		181,300	[35]
Pyridaben	WC	I-21A	1	2012		31		236	[35]
Resmethrin	WC	I-3	5	2010, 2011	8.6	2.4–17	690	150	[35]
Rotenone	P		8	2011, 2012	21.2	1.6–82	300,000	2400	[35]
	WC	I-21B	3	2010	25.5	23–28			
Spinetoram	P	I-5	5	2010, 2011	183.9	4.4–645		7340	[22]
Spiridoclofen	P		4	2012	18.5	5.6–55	1,960,000	2,000,000	[35,37]
	WC	I-23	1	2012		12			
Thiamethoxam	P		5	2009, 2011, 2012	2.8	1.1–3.8	50	240	[35]
	WC	I-4A	4	2010	1.6	1.1–2.8			
**SYNERGIST**									
Piperonyl butoxide	WC		16	2012	15.2	5.3–42		110,000	[35]
	WF	U	3	2011	128.9	6.6–370			

* The parent compound oral and/or contact LD_50_ was used. The parent compound is in parentheses.

**Table 3:** Pesticide detections in pollen by year and pesticide: number of detections (#), and the range and mean (standard deviation (SD)) of the amount detected (in ppb). See Section 3.1, Section 3.3–Section 3.5 for a summary discussion.

	2009			2010			2011			2012		
	#	Range	Mean (SD)	#	Range	Mean (SD)	#	Range	Mean (SD)	#	Range	Mean (SD)
**FUNGICIDES**												
4-Hydroxychlorothalonil	2	5.5–42	23.8 (25.8)	2	5.5–22	13.8 (11.7)	1	21				
Azoxystrobin	3	0.5–56	19.4 (31.7)	12	0.5–214	19.6 (61.2)	15	0.7–14	3.8 (4.0)	11	0.5–37	6.0 (11.0)
Benalaxyl				1	1.9		1	1				
Boscalid				6	1.2–2.8	1.8 (0.6)	4	1.8–5.2	3.7 (1.6)			
Carbendazim	9	0.8–13	4.2 (4.3)	16	1-14	4.0 (3.8)	13	2.1–548	64.7 (146.2)	10	1–56	17.8 (21.5)
Cymoxanil				1	7.5							
Cyprodinil							1	21				
Difenoconazole				1	4.5		1	3.9				
Dimethomorph	1	0.6					1	0.5		1	1	
Fluazinam	1	4.7										
Imazalil (Enilconazole)	1	1.6										
Mandiproamide	1	1.2										
Metalaxyl				1	1.5					3	0.8–3.5	1.9 (1.4)
Metconazole							1	1.3				
Myclobutanil							1	1.1		1	6.1	
Octhilinone							1	24				
Propiconazole-1	2	1.2–3.9	2.6 (1.9)	7	1.1–57	16.5 (19.9)	15	1.1–12	6.7 (6.8)	8	11–64	26.1 (18.5)
Pyraclostrobin				1	12		3	1–3.9	2.1 (1.6)	8	0.8–8.8	3.3 (3.1)
Pyrimethanil				1	29							
Spiroxamine							2	1-2	1.5 (0.7)			
Tebuconazole							7	2–90	27.1 (31.4)	1	104	
Thiabendazole	1	0.8								1	1.5	
Thiophanate-methyl				1	4.6		3	10–40	21.3 (16.3)			
Trifloxystrobin	2	0.2–1.2	0.7 (0.7)	1	23		3	1.4–2	1.7 (0.3)	1	1.6	
**HERBICIDES**												
Acetochlor							1	22		3	1.8–17	7.2 (8.5)
Alachlor	1	1.3										
Atrazine	21	0.1–10	1.9 (2.7)	19	0.2–41	7.4 (10.5)	21	0.2–29	5.4 (8.4)	23	0.4–58	6.8 (14.3)
Bentazon				1	3.1							
Dithiopyr	2	0.6–0.8	0.7 (0.1)	1	3							
Diuron	3	2.4–8.9	5.1 (3.4)	5	4.3–33	13.2 (11.6)	12	3.1–275	48.6 (73.9)	7	1.1–27	11.2 (10.1)
Fenuron				2	0.7–2.1	1.4 (1.0)						
Linuron				2	2.2–14.4	8.3 (8.6)	1	2				
Metolachlor	8	0.2–8.3	2.1 (2.6)	5	5.5–130	48.9 (49.2)	10	0.6–31	6.01 (9.6)	9	0.7–20	4.3 (6.7)
Metribuzin							3	0.6–2.6	1.4 (1.1)			
Metsulfuron-methyl							1	7				
Pendimethalin	13	1.2–14	5.2 (4.8)	11	1-26	9.2 (8.7)	4	2.1–4.1	2.8 (0.9)	13	1.6–55	9.0 (14.1)
Prodiamine	1	2.3										
Prometon							3	1–3.3	2.4 (1.2)			
Prometryn							1	2.2				
Propyzamide										3	1.5–6.2	3.2 (2.6)
Sethoxydim				1	23		2	1.7–2.5	2.1 (0.6)			
Siduron										3	5.6–13	9.1 (3.7)
Simazine	1	1.1					1	2		1	1.4	
Sulfentrazone							1	2.5				
**INSECTICIDES**												
Acephate										2	1.6–3.2	2.4 (1.1)
Acetamiprid				2	10–436	223.0 (301.2)	1	4.8				
Aminocarb							1	1.7				
Amitraz-Metab. DMPMF				1	2.5		1	2.5				
Azinphos-methyl	1	0.4		1	1.1		1	3.5				
Bendiocarb				3	1.2–1.9	1.5 (0.4)						
Carbaryl	3	1.5–8.3	5.1 (3.4)	13	1–6.1	2.9 (1.5)	11	1–95	14.8 (27.3)	16	2.1–21	5.5 (4.4)
Carbofuran	2	1–6.7	3.8 (4.0)	3	12-24	18.0 (6.0)	1	2				
Chlorantraniliprole				1	9.6		1	5.5				
Chlorpyrifos	10	0.9–27	10.4 (11.1)	5	2–10.6	5.4 (3.2)	11	1.6–61	15.5 (18.7)	10	1.4–34	12.8 (11.1)
Clothianidin	1	2		1	1.2					4	1.3–4.8	2.8 (1.7)
Coumaphos	17	0.2–196	20.3 (52.6)	5	1.3–33	8.8 (13.6)	3	1.3–20	8.4 (10.1)	6	1.6–6.1	3.1 (1.7)
Coumaphos oxon										1	2.5	
Diazinon							3	1–8.5	5.1 (3.8)	1	8.6	
Dichlorvos				1	35					1	1.5	
Diflubenzuron							1	19				
Dimethoate	8	0.3–181	27.3 (62.6)	5	0.7–79	18.6 (33.8)	7	2.5–12	7.2 (3.5)	7	0.7–194	33.6 (71.1)
Fenpyroximate	2	0.2–0.6	0.4 (0.3)									
Fluvalinate	7	0.9–4.7	2.5 (1.3)	2	21–27	24.0 (4.2)	3	1.6–2.8	2.4 (0.7)			
Imidacloprid	2	1.5–2	1.8 (0.4)				4	4.4–51	17.8 (22.2)	3	1.8–4.2	2.7 (1.3)
Imidacloprid, Olefin							1	9.5				
Imidacloprid, urea							1	19				
Malathion	1	7.9		9	1.4–138	32.8 (42.0)	11	3.8–397	168.1 (160.1)	12	5.5–148	54.5 (45.6)
Methiocarb				1	2		1	1		5	1.9–20	8.2 (7.8)
Methomyl							3	2.5–26	13.2 (11.9)	3	1.4–19	7.5 (10.0)
Methoxyfenozide										4	2.2–162	45.3 (77.9)
OH-Carbofuran	5	1.2–29	15.8 (11.2)				1	1.9				
Omethoate							6	1–2.4	1.9 (0.6)	3	1-12	6.1 (5.5)
Phorate	1	22										
Phorate Sulfoxide							1	1.7–9	3.3 (3.2)			
Phosmet	4	2.4–57	20.7 (25.1)	4	2.1–483	125.1 (238.6)	5	1.6–82	32.2 (39.4)	4	1.1–26	7.8 (12.1)
Rotenone							5	1.6–82	32.2 (39.4)	3	2.3–16	10.1 (7.0)
Spinetoram				1	4.4		4	8.8–645	363.4 (311.7)			
Spiridoclofen										4	5.6–55	18.5 (23.3)
Thiamethoxam	1	2.8					1	3.1		3	1.1–3.8	2.4 (1.4)

**Table 4:** Pesticides in wax from active colonies: number of detections (#) and amount detected—highest, lowest and mean (SD) from hives (1) Established Spring 2009 and Sampled Fall 2010 [one hive in Maine established in 2010, in brackets and marked by *], (2) Established in Spring 2009 and Sampled Fall 2011 (3) Established Spring 2011/ Sampled Fall 2011 and (4) Established in Spring 2011 and Sampled Fall 2012.

	(1) Established Spring 2009/Sampled Fall 2010 * (n = 52)			(2) Established Spring 2009/Sampled Spring 2011 (n = 18)			(3) Established Spring 2011/Sampled Fall 2011 (n = 7)			(4) Established Spring 2011/Sampled Fall 2012 (n = 65)		
	#	Range	Mean (SD)	#	Range	Mean (SD)	#	Range	Mean (SD)	#	Range	Mean (SD)
**FUNGICIDES**												
4-hydroxychlorothalonil	7	1.4–17	7.3 (5.3)									
Azoxystrobin	4	1–1.3	1.1 (0.2)	4	2.5–3.5	2.9 (0.4)				48	0.5–33	4.9 (6.8)
Boscalid	12	0.6–22	3.9 (5.9)	1	10					1	43	
Carbendazim	6	0.7–56	14.6 (21.2)	9	64–137	89.9 (48.6)	7	1.2–152	101 (49.9)	18	1.5–109	40.6 (28.7)
Diphenylamine				3	25–227	100.7 (110.1)						
Epiconazole	1	0.5										
Fluoxastrobin										1	13	
Mandiproamide	1	2.9										
Myclobutanil	1	4.1								1	1.7	
Octhilinone	3	12-14	13 (1)	4	1.2–4.2	2.9 (1.3)						
Propiconazole-1	13	0.8–66	2.5 (1.5) [1.6] *	4	4.8–8	6.7 (1.4)				19	6.2–27	14.4 (6.9)
Pyraclostrobin				2	1.2–2.1	1.6 (0.6)				51	1.2–47	7.8 (9.4)
Tebuconazole	2	1.3–2.8	2.1 (1.1)	7	1.9–21	10.8 (7.6)	7	1–23.1	10.8 (7.6)	16	2.7–24	6.9 (5.6)
Thiabendazole	7	0.4–92	44.6 (37.0)	1	91					1	2.8	
**HERBICIDES**												
Atrazine	17	0.01–6.5	1.2 (1.5)	6	0.4–2.1	1.0 (0.6)	3	0.5–1.6	1.0 (0.3)	23	0.2–5.4	1.3 (1.2)
Bentazon										19	1.3–17	5.9 (4.8)
Dithiopyr	1	1.8								3	1.7–5.1	3.0 (1.9)
Diuron	9	0.4–3.3	1.4 (0.8)	9	6.4–30	18.1 (8.4)	7	1.7–29.4	16.1 (8.4)	16	2.2–20	7.1 (5.7)
Fenuron				2	24–35	29.5 (7.8)						
Halosulfuron-methyl	3	213–386	307 (87.5)									
Linuron	3	1–2.4	1.5 (0.8)	2	1–5.5	3.3 (3.2)						
MCPA										1	4	
Metolachlor				1	0.7					11	1–4.5	2.2 (1.1)
Napropamide				1	2.8							
Pendimethalin	18	0.4–5.9	2.1 (1.5)							10	2.8–20	6.5 (5.6)
Prometon										1	3.5	
**INSECTICIDES**												
Acephate	1	5.8										
Amitraz Metab DMPF	1	4.6		1	3.7							
Azinphos-methyl	8	0.2–0.3	0.3 (0.1)									
Bendiocarb	1	4.5										
Carbaryl	1	0.9										
Carbofuran	10	0.8–10.8	5.7 (4.0)									
OH-Carbofuran	15	5.4–112	28.1 (34.6)									
Chlorpyrifos	2	0.8–5.5	3.2 (3.3)	9	3.1–15	6.8 (3.6)	7	1.6–14.3	6.6 (4.2)	8	1.4–44	9.5 (14.0)
Coumaphos	32	0.2–75	24.6 (17.8) [17] *	18	8.5–87	25.8 (19.2)	7	8.5–40.1	19.8 (11.8)	65	1.8–2060	62.8 (288.9)
Coumaphos—oxon	21	0.9–5.8	3.1 (1.6) [1.8] *	6	1-12	3.3 (4.3)				11	1.1–43	8.6 (15.1)
Diazinon				6	0.4–2	0.8 (0.6)	5	0.1–1.6	0.8 (0.7)			
Dichlorvos	2	2–2.3	2.2 (0.2)	3	2.3–13	6.2 (5.9)	1	2.3				
Dimethoate	20	1.1–945	63.9 (208.0)	2	0.6–1.9	1.3 (0.92)				25	0.8–52	6.4 (10.2)
Fenpyroximate	1	1.9		4	1–207	72.1 (97.2)	2	1–1.3	1.2 (0.2)	3	5.5–26	17.8 (10.9)
Fluvalinate	24	1–862	348.1 (223.4) [15] *	18	7.6–512	169.3 (151.3)	7	63–407	202.6 (136.1)	62	1.7–537	52.5 (94.9)
Malathion	2	35/49	42 (9.9)	6	1.2–85	26.8 (30.2)	4	11.9–31.2	18.6 (8.8)	17	4.7–91	50.5 (25.7)
Phosmet	18	1.1–220	19.0 (51.0)	1	6.5					11	1.5–32	12.7 (10.5)
Pyridaben										1	31	
Resmethrin	3	6.3–17	13.4 (6.2)	2	2.4–5.5	3.9 (2.2)						
Rotenone	2	23–28	25.5 (3.5)									
Spiridoclofen										1	12	
Thiamethoxam	4	1.1–2.8	1.6 (0.8)									
**SYNERGIST**												
Piperonyl butoxide	21	5.3–53	20.52 (13.8)	4	3.5–37	18.1 (13.9)	7	7.1–22.9	11.4 (7.4)	14	1.7–35	15.7 (11.8)

**Table 5:** Pesticides in 2009 and 2011 Foundation Wax: number of detections, highest, lowest amount detected (ppb).

	2009 (n = 2)			2011 (n = 3)		
	Number of Detections	Amount Detected		Number of Detections	Amount Detected	
		Lowest	Highest		Lowest	Highest
**FUNGICIDES**						
Azoxystrobin	2	2.3	3.4	2	1.8	3.9
Boscalid	2	2.2	6.5	3	3	10
Carbendazim	1		1			
Cyprodinil	1		90	2	8.7	19
Dimethomorph				1		3.9
Fenbuconazole	1		2.5			
Iprodione	2	110	125	1		165
Metalaxzyl				1		1.5
Octhilinone				2	3.1	6.8
Propiconazole-1	1		2.6			
Trifloxystrobin	2	1.3	2.4			
**HERBICIDES**						
Pendimethalin	2	11	17	2	17	17
Prodiamine	2	13.4	17			
Prometon				1		0.5
Prometryn	1		0.5			
**INSECTICIDES**						
Amitraz Metab DMPF	2	510	670	3	16.5	567
Chlorpyrifos	1		17	3	15	28
Coumaphos	2	1584	2500	3	109	1992
Coumaphos oxon	2	48	47	3	6.3	63
Diazinon	1		0.3			
Dichlorvos				1		10
Diflubenzuron				1		3.8
Fenpyroximate	2	4.1	5.5	2	18	366
Fluvalinate	2	7851	15,400	3	290	8718
Propargite	2	31	60	2	19	20
**SYNERGIST**						
Piperonyl butoxide				3	6.6	370

**Table 6:** Mean number of distinct residue detections per pollen sample by year, aggregated across all locations.

Year	Mean # Detections/(Pooled Sample)	Standard Deviation
2009	3.6	2.20
2010	3.6	2.06
2011	4.4	3.06
2012	4.7	3.20

**Table 7:** Most common pesticides detected in pollen samples broken down by year. See Section 3.6.1 for discussion. Pesticides are labeled (F) for fungicides, (H) for herbicides, and (I) for insecticides.

Year	# Pollen Samples	Most Common Pesticides	
		Pesticide	# Sample Detections
2009	34	Atrazine (H)	21 (62%)
		Coumaphos (I)	17 (50%)
		Chlorpyrifos (I)	10 (29%)
2010	41	Atrazine (H)	19 (46%)
		Carbendazim (F)	16 (39%)
		Carbaryl (I)	13 (32%)
2011	51	Atrazine (H)	21 (41%)
		Azoxystrobin (F)	15 (29%)
		Propiconazole (F)	15 (29%)
2012	42	Atrazine (H)	23 (55%)
		Carbaryl (I)	16 (38%)
		Pendimethalin (H)	13 (31%)

**Table 8:** Pesticide MOA for compounds detected in pollen and wax.

CLASS	MODE OF ACTION	PESTICIDES DETECTED
**Fungicides**		
A1	Nucleic acid synthesis: RNA polymerase I	benalaxyl, metalaxyl
A3	Nucleic acid synthesis: DNA/RNA synthesis (proposed)	octhilinone
B1	Mitosis and cell division: β-tubuline assembly in mitosis	carbendazim, thiabendazole, thiophanate-methy
C2	Respiration: Complex II: succinate-dehydrogenase	boscalid
C3	Respiration: Complex III: cytochrome bc1	azoxystrobin, fluoxastrobin, pyraclostrobin, trifloxystrobin,
C5	Respiration: Oxidative phosphorylation uncoupler	fluazinam
D1	Amino acid and protein synthesis: Methionine biosynthesis (proposed)	cyprodinil, pyrimethanil
E	MAP/Histidine-kinase in osmotic signal transduction	iprodione
G1	Sterol biosynthesis in membranes: C14-demthylase in sterol biosynthesis	difenoconazole, epoxiconazole, fenbuconazole, imazali, metconazole, myclobutanil, propiconazole-1, tebuconazole
G2	Sterol biosynthesis in membranes: ∆^14^-reductase and ∆^8^-∆^7^-isomerase in sterol biosynthesis	spiroxamine
H5	Cell wall biosynthesis: Cellulose synthase	dimethomorph, mandipropamid
Multi-site	Multi-site contact activity	chlorothalonil
Unknown	Mode of action is unknown	cymoxanil, diphenylamine
**Herbicides**		
A	Inhibition of acetyl CoA carboxylase	sethoxydim
B	Inhibition of acetolactate synthase ALS	halosulfuron-methyl, metsulfuron-methyl
C1-C3	Inhibition of photosynthesis at photosystem II	C1: atrazine, metribuzin, prometon, prometryn, simazine C2: diruon, fenuron, linuron, siduron C3: bentazon
E	Inhibition of protoporphyrinogen oxidase	sulfentrazone
K1	Mitosis inhibition: Microtubule assembly inhibition	dithiopyr, pendimethalin, prodiamine, propyzamide
K3	Mitosis inhibition: Inhibition of very long chain fatty acid synthesis (inhibition of cell division)	acetochlor, alachlor, metolachlor, napropamide
O	Synthetic auxins	MCPA
**Insecticides**		
1A	Nerve action: Reversible acetylcholinesterase inhibition	aminocarb, bendiocarb, carbaryl, carbofuran, methiocarb, methomyl
1B	Nerve action: Irreversible acetylcholinesterase inhibition	acephate, azinphos-methyl, chlorpyrifos, coumaphos, diazinon, dichlorvos, dimethoate, malathion, omethoate, phorate, phosmet
3	Nerve action: Sodium channel modulator	fluvalinate, resmethrin
4A	Nerve action: Nicotinic acetylcholine receptor agonist	acetamiprid, clothianidin, imidacloprid, thiamethoxam
5	Nerve action: Nicotinic acetylcholine receptor allosteric activator	spinetoram
12C	Inhibitor of mitochondrial ATP synthase	propargite
15	Inhibition of chitin biosynthesis, type 0	diflubenzuron
18	Growth regulation: Ecdysone receptor agonist	methoxyfenozide
19	Nerve action: Octopamine receptor agonist	amitraz
21	Energy metabolism: Mitochondrial complex I electron transport inhibition	A: fenpyroximate, pyridaben B: rotenone
23	Lipid synthesis and growth regulation: Acetyl CoA carboxylase inhibition	spiriclofen
28	Nerve and muscle action: Ryanodine (calcium) receptor modulator	chlorantraniliprole
Unknown	Piperonyl butoxide—synergist, Cytochrome P450-dependent monoxygenase inhibitor	piperonyl butoxide

**Table 9:** Bivariate association (co-occurrence) among different pesticide MOA occurring in pollen.

PESTICIDE INTERACTION	ASSOCIATION		R ^1^	*p*
	MOA CLASS	MOA CLASS		
Fungicide & Fungicide	Fungicide	Fungicide		
	A1	H5	0.4802	0.0188
	B1	G2	0.8986	<0.0001
	B1	Multiple targets	0.4514 *	0.0266
	C3	G1	0.4362	0.0332
Herbicide & Herbicide	Herbicide	Herbicide		
	C1	K1	0.7488	<0.0001
Insecticide & Insecticide	Insecticide	Insecticide		
	A1	3	0.6645	0.0004
	A1	19	0.4041	0.0507
	3	19	0.6575	0.0005
	4A	21B	0.5302	0.0077
	21B	28	0.7170	<0.0001
Fungicide & Herbicide	Fungicide	Herbicide		
	A1	C1	0.7754	<0.0001
	A1	K1	0.4523 *	0.0265
	B1	C2	0.9660	<0.0001
	C2	A	0.6728 *	0.0003
	C3	A	0.9561	<0.0001
	G1	A	0.4387 *	0.0320
	D1	C1	0.9955	<0.0001
	G1	C2	−0.4366 *	0.0338
Fungicide & Insecticide	Fungicide	Insecticide		
	A1	1A	0.4817	0.0171
	A1	4A	0.4054 *	0.0494
	C2	21B	0.7289	<0.0001
	C2	28	0.9910	<0.0001
	C3	28	0.4311 *	0.0354
	G1	28	0.4328	0.0346
	C3	1B	0.4255 *	0.0382
	D1	1B	0.4153 *	0.0436
	G2	1B	0.7726	<0.0001
	G2	4A	0.5213	0.0090
	H5	1A	0.4794 *	0.0178
Herbicide & Insecticide	Herbicide	Insecticide		
	A	28	0.9962 *	<0.0001
	C1	4A	0.6761 *	0.0003
	C2	1B	0.8857	<0.0001
	K1	1A	0.6629	0.0004
	K1	1B	0.5345 *	0.0071
	K1	3	0.7512	<0.0001
	K1	19	0.4628	0.0228

^1^ Default is Pearson correlation coefficient; Spearman’s rank correlation coefficient is indicated by *.
